# Natural and glucosyl flavonoids inhibit poly(ADP-ribose) polymerase activity and induce synthetic lethality in BRCA mutant cells

**DOI:** 10.3892/or.2013.2902

**Published:** 2013-12-05

**Authors:** JUNKO MAEDA, ERICA J. ROYBAL, COLLEEN A. BRENTS, MITSURU UESAKA, YASUSHI AIZAWA, TAKAMITSU A. KATO

**Affiliations:** 1 Department of Environmental and Radiological Health Sciences, Colorado State University, Fort Collins, CO 80523, USA; 2 Graduate School of Engineering, The University of Tokyo, Tokyo 113-8656, Japan; 3 Research and Development Group, Toyo Sugar Refining Co., Ltd., Tokyo 103-0046, Japan

**Keywords:** flavonoid, BRCA2, PARP inhibitor

## Abstract

Poly(ADP-ribose) polymerase (PARP) inhibitors have been proven to represent superior clinical agents targeting DNA repair mechanisms in cancer therapy. We investigated PARP inhibitory effects of the natural and synthetic flavonoids (quercetin, rutin, monoglucosyl rutin and maltooligosyl rutin) and tested the synthetic lethality in BRCA2 mutated cells. *In vitro* ELISA assay suggested that the flavonoids have inhibitory effects on PARP activity, but glucosyl modifications reduced the inhibitory effect. Cytotoxicity tests of Chinese hamster cells defective in BRCA2 gene (V-C8) and its parental V79 cells showed BRCA2-dependent synthetic lethality when treated with the flavonoids. BRCA2 mutated cells were three times more sensitive to the flavonoids than the wild-type and gene complemented cells. Reduced toxicity was observed in a glucosyl modification-dependent manner. The present study provides support for the clinical use of new treatment drugs, and is the beginning of the potential application of flavonoids in cancer prevention and the periodic consumption of appropriate flavonoids to reduce cancer risk in individuals carrying a mutant allele of the BRCA2 gene.

## Introduction

**Figure 1:**
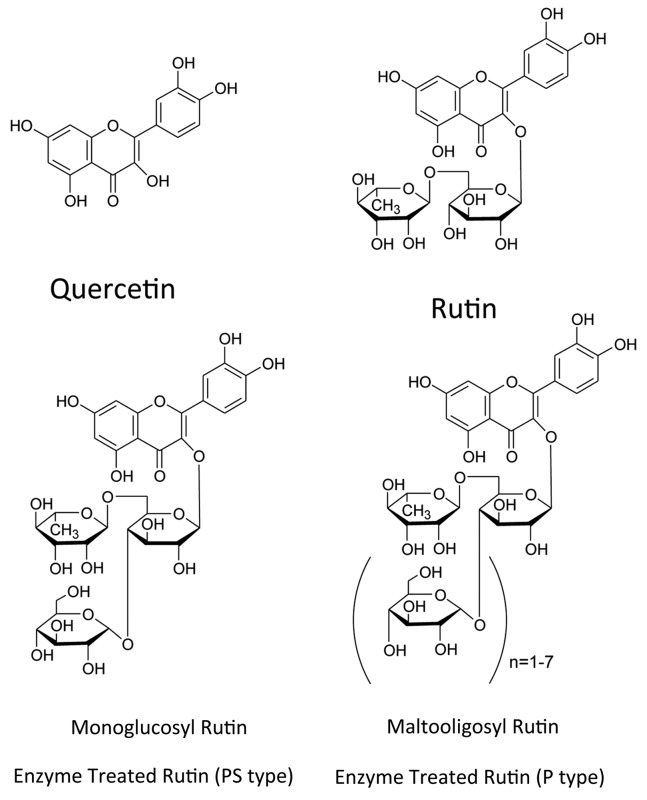
Chemical structures of flavonoids. Adding glucosyl residues to quercetin cores produces natural and synthetic flavonoids.

The natural flavonoids quercetin and rutin, and synthetic flavonoid monoglucosyl rutin (alpha-glucosyl rutin™) are commercially available and widely utilized in our daily diet. Quercetin, the representative of the flavonol subclass of flavonoids, remains a key portion of the human diet occurring in fruits, vegetables, leaves and grains (1,2). Quercetin and rutin are also used as a dietary antioxidant supplement in food and beverages (3). Rutin has two glucosyl residues with quercetin, monoglucosyl rutin has three glucosyl residues, and maltooligosyl rutin has four to seven glucosyl residues (Fig. 1) (4). In addition to the antioxidant activity, quercetin and rutin have been identified to have multiple inhibitory effects in mammalian cells with quercetin being used for a variety of beneficial health effects including the treatment of heart conditions, inflammation and diabetes (5–7). Moreover, the role of the flavonoid potential in the antitumor effect and cancer prevention has been studied against various types of cancers (8). Through investigating apoptotic effects of quercetin in human myeloid leukemia cells, this flavonoid proved to induce the poly(ADP-ribose) polymerase (PARP) inhibitory effect (9).

Synthetic lethality where cell death occurs due to the combination of a gene and small molecule has been a focus in cancer therapy (10). Studies have suggested synthetic lethal interaction between PARP inhibition and BRCA deficiency (11). *In vitro* and *in vivo* evidence has supported the utilization of PARP inhibitors as single agents to reduce cancer cells that possess a defect in DNA repair that additionally have BRCA1 and BRCA2 mutations (12). PARP is an enzyme to form large poly(ADP-ribose) polymers in live cells that have DNA damage (13). PARP plays a significant role in single strand DNA break repair that leads to no repair occurring if there is a defect in homologous recombination repair (HRR) present with synthetic lethality of PARP inhibitors in HRR-defective cells. PARP inhibitors render HRR-defective cells unable to maintain their genome during replication and die (14,15). Currently, more than five PARP inhibitors are employed in clinical trial development (16).

Heterozygous mutation of BRCA1 or BRCA2 causes hereditary breast and ovarian cancer syndromes at an early age and increases the chance of bilateral cancers (17,18). This gene mutation is transmitted in an autosomal dominant pattern in families (19). In the general population this gene mutation is rare; in the US, one in 400–800 individuals have the BRCA1 or BRCA2 heterozygous mutations (20). However, BRCA1 and BRCA2 mutations are dangerous mutations that produce a hereditary breast ovarian cancer at an estimation of 40.7% in these mutant carriers (21,22). The focus of the present study was the impact of flavonoids on PARP when combined with BRCA mutated cells. We tested the activities of PARP inhibition with variations of glucosyl modifications of quercetin by analyzing PARP inhibition assay *in vitro* and poly(ADP-ribose) immunostaining. We investigated the existence of synthetic lethality between BRCA2 mutants and flavonoids through the cytotoxicity assay along with the γ-H2AX foci experiment.

## Materials and methods

### Cell culture

Chinese hamster ovary (CHO) cells, Chinese hamster lung V79 cells wild-type and its BRCA2 mutant (V-C8) (23), and V-C8 hBRCA2, genetically complimented mutant with human BRCA2 cDNA were cultured in Minimum Essential Medium (MEM)-α (Gibco, Indianapolis, IN, USA) and supplemented with 10% fetal bovine serum (FBS; Sigma, St. Louis, MO, USA) and 1% antibiotics and antimycotics (Gibco). They were maintained at 37°C in a humidified atmosphere of 5% CO_2_ in air.

### Chemicals

Quercetin, rutin, monoglucosyl rutin and maltooligosyl rutin were provided from Toyo Sugar Refining Co., Ltd. (Tokyo, Japan) (Fig. 1). AlphaGrutinPS™ and alphaGrutinP™ are trademarks of Toyo Sugar Refining. Quercetin was prepared in dimetyl sulfoxide (DMSO) for a 1% (w/v) solution. Rutin was prepared in DMSO for 10% (w/v) solution. Monoglucosyl rutin and maltooligosyl rutin were prepared in phosphate-buffered saline (PBS) for 10% (w/v) solution. For the positive control, 3-aminobenzamide (Trevigen, Gaithersburg, MD, USA) was utilized. The concentrations testing in the present study were in the range from 0.00001 to 1% (w/v).

### PARP inhibitor assay in vitro

PARP inhibitory ELISA kit from Trevigen was used for the present study (24). PARP was incubated in a 96-well microplate with a reaction mixture containing 50 μM β-NAD^+^ (10% biotinylated β-NAD^+^), 90% unlabeled β-NAD^+^, 1 mM 1,4-dithiothreitol and 1.25 mg/l nicked DNA. The formation of the poly(ADP-ribose) polymers was detected with peroxidase-labeled streptavidin (Invitrogen, Grand Island, NY, USA) and 3,3′,5,5′-tetramethylbenzidine. PARP-1 activity was expressed as absorbance at 450 nm. PARP-1 inhibition of flavonoids was evaluated by addition of these compounds to the reaction mixture. NanoDrop spectrophotometer (Thermo Fischer Scientific, Waltham, MA, USA)measured absorbance.

### Poly(ADP-ribose) immunostaining

The suppression of poly(ADP-ribose) formation by flavonoids was investigated by immunostaining. Cultured CHO cells were treated with natural and synthetic flavonoids for 30 min to one day. H_2_O_2_ (2 mM) was added for 10 min before fixation. Cells were fixed in 4% paraformaldehyde for 15 min and treated in 0.2% Triton X-100 solution for 10 min, following overnight blocking in PBS with 10% goat serum. Immunostaining was carried out with anti-poly(ADP-ribose) monoclonal antibody (BD Biosciences, San Jose, CA, USA) (25) with 1:300 dilution. Poly(ADP-ribose) formation was analyzed under Zeiss Axioplan fluorescence microscope (Carl Zeiss, Oberkochen, Germany) with QImaging EXi Aqua digital camera (QImaging, Surrey, BC, Canada).

### Cytotoxicity test by colony formation assay

Exponentially growing cells were trypsinized and plated on P-60 dishes to obtain ~100 colonies per dish. Different concentrations of natural and synthetic flavonoids were added. Ten days later, cells were fixed in 100% ethanol and stained by 0.1% Crystal violet solution (Sigma). Colonies containing >50 cells were counted as survivors.

### γ-H2AX foci formation

Following treatment of V79 and mutant cells with flavonoids, cells were treated as poly(ADP ribose) immunostaining. The primary antibody utilized was mouse monoclonal γ-H2AX antibody (Millipore, Billerica, MA, USA) in 1:300 dilution. Secondary antibodies used were Alexa 488 Fluor-conjugated goat anti-mouse antibody (Invitrogen). DNA was counterstained by DAPI (4′,6-diamidino-2-phenylindole) with Prolong Gold Antifade (Invitrogen). Images were captured and analyzed with Zeiss Axioplan fluorescence microscope with QImaging EXI Aqua digital camera. Nuclei containing >100 foci per cell were scored as massive DNA damage from synthetic lethality.

### Statistics

All experiments were carried out at least three times and error bars indicate standard error of the means. Analysis of variance was used to determine statistical significance with Prism 5 software (GraphPad Software, Inc., La Jolla, CA, USA). For all analyses, P-values of <0.05 were considered to indicate a statistically significant result.

## Results

### In vitro ELISA assay for PARP activity

**Figure 2:**
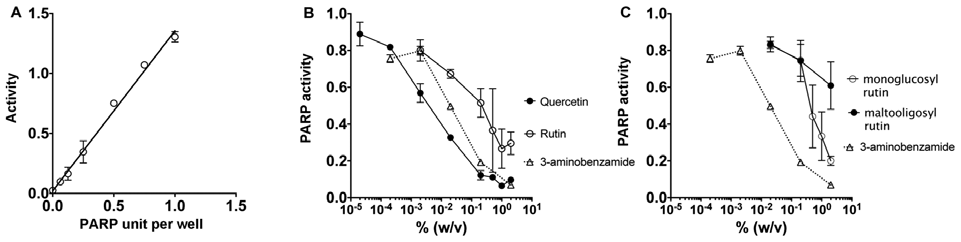

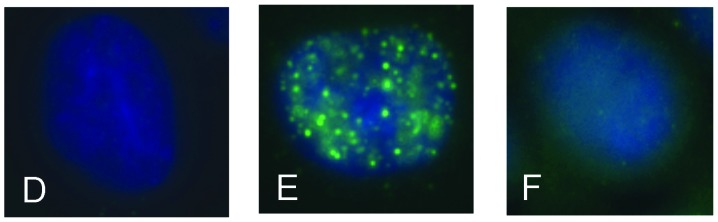
Inhibition of PARP activity by flavonoids. (A–C) *In vitro* PARP activity assay using a PARP activity ELISA kit. (A) PARP standard curve, (B, C) dose-response curves of four flavonoids and a PARP inhibitor 3-aminobenzamide. Three independent experiments were performed. Error bars represent standard error of the mean. (D–F) Representative immunofluorescence images showing inhibition of poly(ADP-ribose) formation by quercetin in CHO cells. Poly(ADP-ribose) (green) and DNA (blue) are shown. An untreated cell is depicted (D), a cell after H_2_O_2_ (2 mM) treatment for 10 min (E), and the same H_2_O_2_ treatment cell following the quercetin (0.01%) pretreatment for 30 min (F).

The *in vitro* assay for PARP activity was carried out with a screening of PARP inhibiting assay through the measurement of polyADP-ribosylation of histone proteins (Fig. 2A–C). 3-aminobenzamide was utilized as the positive control based on its popularity as a PARP inhibitor. Fig. 2A shows the standard curve of the PARP activity that demonstrates a linear relationship with increasing concentration. Fig. 2B and C demonstrate a dose-dependent relationship where an increase in dosage for all four flavonoid drugs and the positive control 3-aminobenzamide results in decreased PARP activity. Quercetin displayed the strongest inhibitory effects on PARP. The two-glucosyl residues that form rutin from the quercetin structure dramatically changed PARP inhibitory effects, however, rutin still carried PARP inhibitory effect. Rutin displayed a greater PARP inhibitory effect than monoglucosyl rutin with three glucosyl residues and maltooligosyl rutin with up to seven glucosyl residues.

### Inhibition of PARP in vivo

The PARP activity inhibition by flavonoids was confirmed by immunostaining to detect poly(ADP-ribose) formation with CHO cells (Fig. 2D–F). Clear poly(ADP-ribose) formation in CHO wild-type cells was observed after 10 min of H_2_O_2_ (2 mM) treatment under a fluorescence microscope (Fig. 2D and E). On the other hand, pretreatment of quercetin [0.01% (w/v), 0.6 mM] inhibited H_2_O_2_ induced poly(ADP-ribose) formation in the CHO cells (Fig. 2F). Although other flavonoids did not suppress poly(ADP-ribose) formation as well as quercetin, overnight pretreatment of rutin, monoglucosyl rutin and maltooligosyl rutin showed inhibition of the poly(ADP-ribose) formation.

### Synthetic lethality in BRCA2 mutant cells with drug treatment

**Figure 3:**
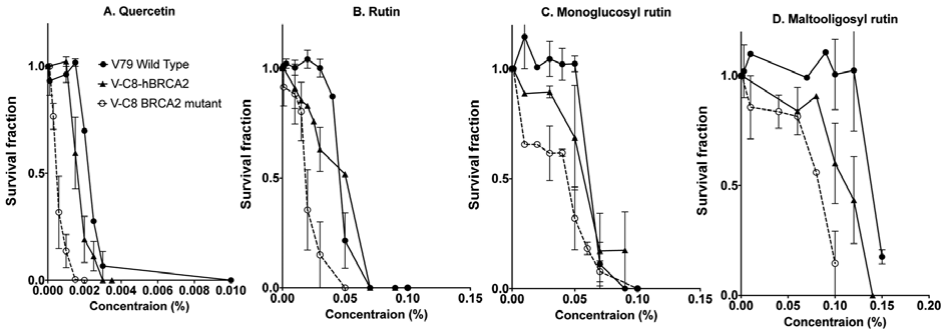
Survival curves of flavonoid-treated BRCA2 mutant and BRCA2 proficient cells. V79 (wild-type; closed circles), V-C8 (BRCA2 mutant; open circles) and V-C8 hBRCA2 (V-C8 complemented with human BRCA2 gene; closed triangles) cells upon continuous exposure to (A) quercetin, (B) rutin, (C) monoglucosyl rutin or (D) maltooligosyl rutin at different concentrations in media. At least three independent experiments were carried out. Error bars represent standard error of the means.

Cytotoxicity studies were carried out with V79 wild-type and its BRCA2 mutant (V-C8) and human BRCA2 gene complimented V-C8 (V-C8 hBRCA2) cells (Fig. 3). Each quercetin and the three variations of glucosyl modifications of rutin, monoglucosyl rutin and maltooligosyl rutin were continuously present in media during colony formation. Quercetin showed the highest cytotoxicity among the four flavonoids. V-C8 cells exposed to flavonoids possessed a higher lethality to the survival fraction with a lower concentration in comparison with V79 wild-type and genetically complimented V-C8 hBRCA2 cells. The concentration required for 90% cell death for quercetin was <0.003% (0.1 mM), rutin was at a concentration of 0.06%, monoglucosyl rutin increased to ~0.075%, and maltooligosyl rutin had the highest concentration at 0.15% for V79 cells. It was also apparent that additional glucosyl residues reduced cytotoxicity for V79 wild-type, V-C8 and V-C8 hBRCA2 cells.

### DNA double strand breaks from synthetic lethality

**Figure 4:**
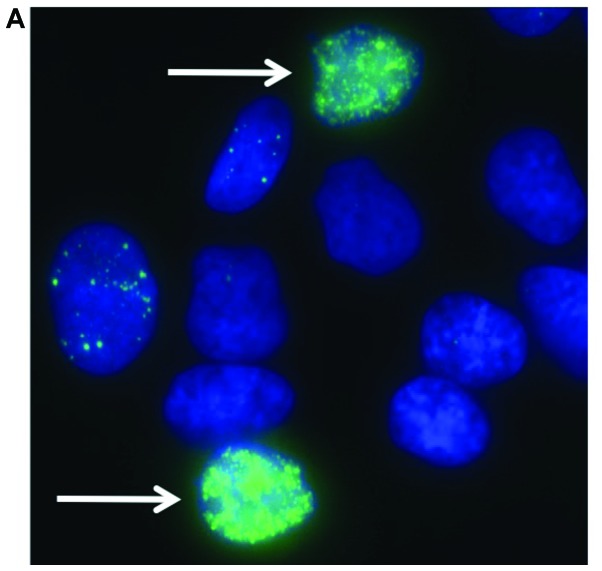

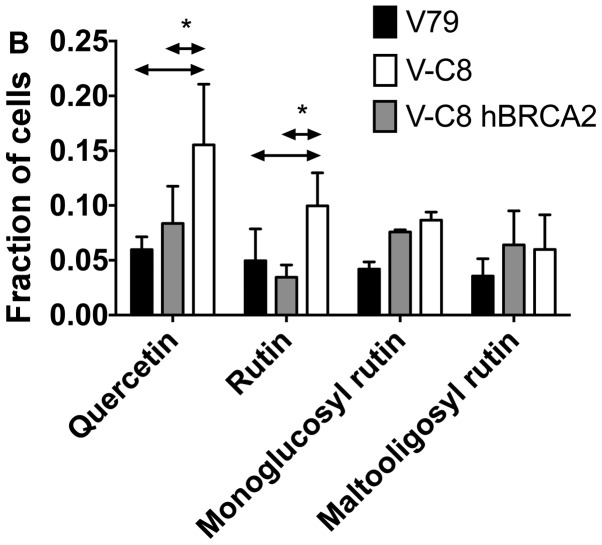
γ-H2AX foci formation induced by synthetic lethality after overnight treatment with four flavonoids (0.01%). (A) Representative image of γ-H2AX foci formation after quercetin treatment in V79 cells. Arrows indicate massive γ-H2AX foci in nuclei. (B) Induced fraction of cells containing γ-H2AX foci after flavonoid treatment in V79, V-C8 and V-C8 hBRCA2 cells. Three independent experiments were carried out. Error bars represent standard error. ^*^P<0.05 by Student’s t-test.

To assess DNA damage from synthetic lethality, we used γ-H2AX foci, a marker of DNA double strand breaks (Fig. 4) (26). V79 wild-type cells, its BRCA2 mutant (V-C8) and genetically complimented mutant with human BRCA2 (V-C8 hBRCA2) were exposed each to 0.01% flavonoid for 12 h. Synthetic lethality induced DNA damage was observed in pan nuclear region and clearly distinguished from background level focus (Fig. 4A). The fraction of population having >100 nuclear foci was scored as massive DNA damage from synthetic lethality (Fig. 4B). The V-C8 cells displayed the greatest fraction of cells displaying massive DNA damage compared with V79 in the four drug treatments. Human BRCA2 complementation to V-C8 cells was visible in the drug treatments of quercetin and rutin. The wild-type cells possessed fewer number of massive DNA damage in the four drug exposures. Quercetin demonstrated the greatest effect regarding DNA damage on the three cell lines. Rutin had a less significant effect in comparison with quercetin in all three cell lines. Monoglucosyl rutin and maltooligosyl rutin did not cause an equivalent amount of damage to cells in comparison with quercetin and rutin.

## Discussion

Our studies showed that the natural and glucosylated flavonoids quercetin, rutin, monoglucosyl rutin and maltooligosyl rutin inhibited PARP activity and induced synthetic lethality in BRCA2 deficient Chinese hamster cells (Figs. 2 and 3). Synthetic lethality was observed through the greatest production of DNA double strand breaks from the combination of BRCA2 deficient V-C8 cells with PARP inhibition through flavonoid exposure in comparison with the other two BRCA2 proficient cell lines (Fig. 4). Quercetin demonstrated the greatest effect regarding PARP inhibition in conjunction with V-C8 cells in comparison with flavonoids that possessed glucosyl modifications, which speaks to the lethality occurring in a glucosyl-dependent manner.

The observed effect of quercetin and glucosyl flavonoids in BRCA2 mutant cells as a result of another pathway than PARP inhibition has to be formally considered. For example, we can not exclude that previous studies have identified flavonoid cytotoxicity on cells as a result of increasing intracellular reactive oxygen species levels or inhibiting DNA topoisomerase (27,28). However, synthetic lethality effect is likely because we confirmed the inhibition of PARP both *in vitro* and in cells, and the flavonoids selectively targeted BRCA2 mutated cells, which is consistent with a cytotoxic mechanism involving the conversion of single strand breaks into double strand breaks during DNA replication that cannot be repaired efficiently in cells with an HRR defect. Thus, the activity of flavonoids was clearly dependent on BRCA2 dysfunction and represents a new clinical application for BRCA2 mutant in breast cancer.

The modification of physiochemical and biological properties of flavonoid structures is of great scientific and industrial interest. Our observations suggest that the efficacy of the flavonoids as PARP inhibitors appears to result from the number of glucosyl residues (Figs. 2A–C and 3). Monoglucosyl rutin and maltooligosyl rutin act as PARP inhibitors, but are not as effective as quercetin. These quercetin glycosides showed higher solubility in water than quercetin due to the hydrophilicity of the sugar moieties, suggesting that the conjugation with glucose enhances quercetin absorption in small intestine (4). In contrast, rutin, which has glycosides, has been found to not incorporate well into the cells (29). One possibility is that the higher hydrophilicity of rutin and glucosylated rutin caused them to be barely incorporated into the cells and, therefore, showed less effect in the present study. It is also possible that glycosyl residues might block PARP inhibitory activity. As we have shown in *in vitro* assay for PARP activity (Fig. 2A–C), quercetin glucosyl derivatives demonstrated a lower PARP inhibitory activity with increased number of glucosyl residues in comparison with quercetin. We assume these additional glucoses would decrease PARP inhibitory activity, however, a longer exposure duration would allow cells to uptake and digest the glucosyl residues.

Although there are many *in vitro* and *in vivo* studies related to flavonoid toxic and mutagenic properties, it remains a vital part of drug exposure to know the possible genotoxicity and cytotoxicity for natural or synthetic flavonoids (1). In earlier studies, the potential mutagenic effects of flavonoids have been reported, including sister chromatid exchanges and bacterial system tests (30,31). Several reports supported the absence of dietary quercetin-related carcinogenicity and toxicity *in vivo* (1). While previous animal studies have demonstrated that following oral consumption of quercetin, the plasma levels of total quercetin (free and conjugated) were quite high (0.05 mM), the free un-conjugated quercetin circulated in plasma at extremely low concentrations. We observed considerable cytotoxicity of quercetin in wild-type cells at relatively high concentrations (0.1 mM) and in a dose-dependent manner. These results are consistent with previous reports of observed flavonoid cytotoxicity in wild-type CHO and non-cancerous human cell lines (27,32). Even better cytotoxicity to BRCA2 mutated cells have been achieved by sufficient active flavonoid concentrations; exposure of high dose of genotoxic flavonoids may have unwanted effects to the healthy tissues.

Currently, oral intake of PARP inhibitors has shown a favorable therapeutic score for breast cancer with acceptably low toxicity in women with the BRCA1 and BRCA2 mutation (33). Moreover, the role of PARP inhibitors for chemoprevention for breast and ovarian cancer in BRCA1 and BRCA2 mutation carriers has been proposed. However, caution should be exercised for long-term use of these drugs in the light of the induction of resistance, and especially for the lack of knowledge of the effects of long-term inhibition of PARP. We propose that the new natural agents may have the potential of reducing cancer risk by the elimination of BRCA1 or BRCA2 homozygous mutated cancers. The present study provides support for the clinical use of dietary flavonoids, and is the beginning of the potential application regarding cancer prevention and potentially periodic consumption of appropriate flavonoids.
